# Dosage

**Published:** 1911-12

**Authors:** George L. Servoss

**Affiliations:** Fallon, Nevada


For Texas Medical Journal.

### Dosage.

BY GEORGE L. SERVOSS, M. D., FALLON, NEVADA.

The subject of the administration of drugs, as regards the amount employed in each individual dose, is a matter which has been under discussion since the giving of medicine has been in vogue. Under the older systems of medicine, drugs were given in large quantities, and at either long or short intervals, according to the indications. Under this system it has been the effort of the physician to obtain full results from the drug at each successive dose. Through having obtained such effect, it is frequently found that a subsequent dose of the drug can not be administered for some time, as a rule, not until the effect of the preceding dose has practically passed over. In consequence of this, when drugs are exhibited in this manner, there is a loss of action, to a greater or less extent, due to the length of the interval.

From the foregoing it would seem that the administration of full physiologic doses at infrequent intervals is faulty to a certain extent. The effect of the drug is not continuous and there is, consequently, a loss of dynamic power, which is most desirable in the majority of instances. When this system is followed, not only is there a loss of drug action during the latter portion of the intervals between doses, but there is a liability that there will be an occurrence of certain undesirable drug actions. Large doses of drugs are not infrequently provocative of the condition known as idiosyncrasy. When large amounts of any drug are given, the effect upon the system is similar to that exerted by the sledge hammer; too heavy blows are frequently struck. Cumulative effect is frequently produced through the continued use of physiologic doses, in that the eliminative organs or channels do not carry off any possible over-amount of the drug employed.

A number of years ago, in Europe, Burggraeve, while experimenting with the alakaloids and other active principles of plant drugs, found that it was possible to obtain therapeutic effect from drugs without the exhibition such agents in individual physiologic doses. In order that he might obtain such results, the agents employed were reduced in dosage to seemingly infinitesimal and inactive amounts, in so far as each individual dose might be concerned. These small amounts were exhibited at very frequent intervals, the physician watching all of the signs and symptoms at all times during such administration. It was found that, in many cases, the therapeutic effect was to be determined prior to the establishment of the full physiologic action. When the therapeutic action was established, the interval between doses was lengthened to such time as was shown that the therapeutic effect was maintained. By this system of dosage it was found that, even though many patients had shown an idiosyncrasy for certain drugs, when given under the dosage of the older system, no such effect was established when minimum amounts were exhibited with the therapeutic and not the physiologic effect in mind. It was also found that even in those persons showing no disposition to an idiosyncrasy, the effect of the agents employed was preferable to that obtaining under the older system.

Under the system of administrating drugs with the idea that the physiologic effect might obtain with each individual dose many undesirable actions were brought about. Nausea and even vomiting not infrequently followed, both of which in many instances, were undesirable. With the use of the later system of small dose, neither of these conditions obtained, as a rule. In fact, it has been found that the therapeutic effect is not infrequently established before sufficient of the drug has been exhibited to produce other than that action.

Not only did Burggraeve find that this system of dosage applied to the active principles of plant drugs, but to the major portion of all other drug agents. He found that drugs otherwise toxic in action, when administered in full physiologic dosage, might be given without fear when employed in very small amounts and at frequent intervals.

This later system of drug application has given the physician a scheme of medication far superior to that of the other style of treatment; one far more rational. By following the plan of Burggraeve and administering small doses at frequent intervals to therapeutic effect and maintaining such effect by lengthening the interval, there is absolutely no loss of the dynamic power of the agent employed, the action going on evenly and unbroken as long as the drug may be exhibited.

Not infrequently is it found that when the drug is divided into the smaller doses and exhibited as outlined above, the total amount employed is far less than under the older system. In some instances the case is carried through to termination with the amount of the drug which would, under the older system, represent but a very few doses.

The Burggraeve, or dosimetric system, is not only preferable because of its more rational features, but also from a point of economy. In the majority of instances far less of the drugs indicated are employed, thereby bringing about a saving in expense to the patient.

The dosimetric system of medication has also served to bring about a condition known as jugulation, whereby acute diseases are either aborted, through prompt and continued drug action, or their severity placed in abeyance, with greater promptness in satisfactory terminations. In numerous instances it has been found that when drugs have been exhibited according to the rules of this system recovery has followed in far shorter than the accepted period and that many of the "self-limited" diseases have lost many of their terrors, both to the patient and doctor alike.

Under the older system, the efficacy of many drugs was questioned. This was due largely to the fact that, under that system of medication, if administered in sufficient quantity to obtain the desired results, there was a liability of undesirable effect. Under the dosimetric system, in which the agents are administered to therapeutic effect only, as a rule, no untoward effects obtained and because of this it has been found that many of the so-called inefficient drugs have been re-established in their remedial positions. Dosimetry has done much to re-establish drug therapeusis, thereby bringing relief to many who otherwise would have been treated under the expectant plan.

